# Epidemiology of Arrhythmias in Children

**Published:** 2008-05-01

**Authors:** R Prem Sekar

**Affiliations:** Consultant Paediatric Cardiologist, Frontier Lifeline Hospital, Chennai, India

**Keywords:** epidemiology, arrhythmias, children

Unlike the adult population, arrhythmias occur less commonly in childhood. Only 5% of the emergency hospital admissions in the paediatric population is attributed to symptomatic arrhythmias [1]. Majority of these tend to be accessory pathway mediated supraventricular tachyarrhythmias such as Wolff-Parkinson-White (WPW) syndrome, permanent junctional reciprocating tachycardia (PJRT) and Mahaim tachycardia [2]. The non accessory pathway mediated supraventricular tachyarrhythmias commonly seen in children are junctional ectopic tachycardia (JET) and automatic ectopic atrial tachycardia (AET) [2] and occur mostly in the post operative period after intracardiac repair for a structural heart defect. Ventricular tachycardia (VT) although uncommon, occurs in the paediatric age group in association with hypertrophic cardiomyopathy (HCM), long QT syndrome (LQTS) and Brugada syndrome. Occasionally, VT can also present symptomatically as incessant idiopathic infant ventricular tachycardia, right ventricular outflow tract tachycardia, benign VT, catecholaminic VT, idiopathic left ventricular tachycardia and in post cardiac surgical patients.

The symptomatology of arrhythmias in children depends on the underlying rhythm disorder and the age at presentation. Typically, neonates and infants with arrhythmias tend to present with congestive cardiac failure secondary to tachycardiomyopathies. This is particularly known to occur following PJRT, AET and VT. Palpitation and or syncope are the mode of presentation in older children. Complete heart block is the bradyarrhythmia of significance seen in childhood. This is either of congenital etiology or a sequela to cardiac surgery. Infrequently, sudden cardiac death (SCD) occurs due to arrhythmias particularly when they are of ventricular origin. SCD of arrhythmogenic etiology in children is reported with HCM, LQTS, Type B WPW syndrome, complete heart block and ventricular arrhythmias post cardiac surgery.

## Fetal Tachyarrhythmias

Atrioventricular reentry (AVRT) remains the most common mechanism for fetal tachyarrhythmias. Infrequently atrial reentry tachycardias are seen. With sustained tachycardia there is a 50% risk of the fetus developing congestive cardiac failure and 50% risk of fetal death with untreated hydrops [3]. Sustained tachycardia and lower gestation at presentation are associated with a higher risk of congestive cardiac failure. The treatment options following diagnosis of fetal tachycardia include observation, inducing delivery if gestation is sufficiently advanced, transplacental drug therapy or direct fetal therapy by the intra-amniotic, intra-peritoneal, fetal intramuscular or fetal intravenous routes. Maternal oral administration of digoxin, flecainide or both has been the widely tried and successful method of managing fetal tachyarrhythmias.

## Supraventricular Arrhythmias

The supraventricular tachycardia (SVT) occurring in earlier life is commonly accessory pathway mediated. Atrioventricular reciprocating tachycardia (AVRT) represents 85% of the arrhythmias in fetal life and 82% of the arrhythmias occurring during infancy. In most cases tachycardia will resolve spontaneously by the end of infancy although there may be late recurrences [4]. The incidence decreases to 65% in the 1-5 age group, 56% in the 6-10 age group and 68% in the above 10 years age group. Atrioventricular nodal reentrant tachycardia (AVNRT) is uncommon during infancy accounting only for 4% of the arrhythmias. The incidence of this is about 23% in the 1-5 age group, 34% in the 6-10 age group and 20% in those over 10 years of age. Most of these do not resolve spontaneously and will require radio frequency (RF) ablation [5]. Less common arrhythmias include atrial flutter (AF), chaotic atrial tachycardia and atrial ectopic tachycardia. The incidence of atrial tachycardia is around 10-15% during childhood and most resolve spontaneously. When persistent, RF ablation is curative [5].

***Wolff-Parkinson-White (WPW) syndrome*** is a typical example of AVRT in children. The population prevalence of ventricular pre-excitation is around 1.5 per 1000 in the adolescent age group and probably lower during the early childhood [6]. A significant proportion of patients do not reveal pre-excitation in the ECG at the time of presentation. Nearly 50% remain asymptomatic at diagnosis with 30% having symptoms due to arrhythmias at a later time [6]. The overall risk of SCD in WPW syndrome is low and is estimated at 2 per 1000 patient years [7]. SCD in WPW is probably due to ventricular fibrillation which is secondary to atrial fibrillation. The main predictor of risk of SCD is symptoms. A patient who presents with syncope or a patient previously resuscitated from cardiac arrest poses a higher risk. The other risk factors are multiple accessory pathways, associated Ebstein's anomaly of the tricuspid valve and familial pre-excitation. High-risk patients typically reveal a short refractory period of less than 220 ms during electrophysiological study. Noninvasively, the refractory period can only be measured if there is spontaneous atrial fibrillation. Shorter the RR interval, higher the risk of syncope or SCD. A right posteroseptal pathway on a resting ECG is also identified with WPW syndrome patients who have been resuscitated from VF and is considered a high risk for SCD. In contrast, sudden loss of pre-excitation on an exercise ECG and intermittent or alternating pre-excitation on ambulatory ECG monitoring imply a long refractory period and a lower risk of associated SCD [8]. Radio frequency ablation is the treatment of choice in symptomatic patients with WPW syndrome [9].

***Permanent Junctional Reciprocating Tachycardia*** is the commonest form of incessant supraventricular tachycardia in children. This is an orthodromic AVRT with RP>PR and presents typically at 3-4 years of age. These are likely to persist for a long time and are known to cause tachycardiomyopathy. Although pharmacological control is possible with amiodarone or verapamil or in combination with digoxin, the best long-term management is reportedly by RF ablation [10].

***Atrial tachycardias*** in childhood are commonly seen as a result of postoperative atrial scarring, distortion of anatomy, changes in the wall stress and changes in the atrial refractoriness associated with sinus node dysfunction. Atrial tachycardia with one to one conduction can cause SCD. In a collaborative study of atrial flutter of 380 cases by electrophysiological study, significantly more young people with atrial flutter died when no drug control was achieved, compared to those with pharmacologically controlled atrial flutter. Surgical intervention to improve the cardiac haemodynamic function showed a significant decrease in the incidence of atrial flutter and this should be considered whenever feasible [11]. Atrial fibrillation is very rare in infants and children. The atrial rate tends to be faster, usually between 350-400 per minute. Most newborns with atrial fibrillation have a structurally normal heart. When an underlying cardiac pathology exists, it is usually a condition with enlarged right atrium such as Ebsteins anomaly of tricuspid valve or tricuspid atresia. When detected antenatally, medical management of the atrial fibrillation is warranted to prevent hydrops fetalis. 30% of children with atrial fibrillation tend to remain asymptomatic while 30% present with congestive cardiac failure, 25% with palpitations and 15% with syncope.

## Ventricular Arrhythmias

Ventricular premature contractions (VPC) are the commonest ventricular conduction anomaly seen in neonates. Up to 33% of neonates have VPCs in the first week of life and these usually resolve by two weeks of age. However, if they persist beyond this period then further investigation is warranted to rule out an underlying structural heart defect. Ventricular arrhythmias are rare in childhood and may be benign or malignant. Benign VT is a condition diagnosed by exclusion and these children usually have normal ECG, chest X-Ray, and echocardiography and in some cases when performed, a normal EP study. The VT is suppressed by exercise and is usually refractory to medical management with a good long-term prognosis. Incessant idiopathic infant VT presents in the first two years of life with a male preponderance and is considered to be secondary to a left ventricular hamartoma. The children present with CCF or collapse. The VT best responds to pharmacological treatment with flecainide and amiodarone and usually resolves spontaneously by school age facilitating withdrawal of drugs. RVOT tachycardia is occasionally seen in teenagers and is distinct from arrhythmogenic right ventricular dysplasia or cardiomyopathy. The ECG shows LBBB with a vertical or right axis. This VT is usually produced by exertion or emotion, can be reproduced by isoprenaline or burst pacing and is amenable to RF ablation. Idiopathic LV tachycardia is rare and arises from the posterior fascicle of the left bundle branch and is thought to originate from the Purkinje network. It responds to treatment with verapamil and is also amenable to RF ablation. Catecholaminergic VT, first described by Coumel, is induced by emotion or exertion and can result in torsades associated with syncope. The resting ECG is normal. Isoprenaline administration or exercise testing can reproduce the VT. This is a dangerous form of VT and the prognosis is greatly improved by beta blocker therapy [12].

***Long QT syndrome (LQTS)*** is a familial disease characterized by prolonged and abnormal ventricular repolarisation and by the risk of life threatening ventricular arrhythmias. Recent molecular genetic studies in LQTS families have identified aberrations in genes encoding for cardiac ion channels. Dysfunction of these cardiac ion channels leads to prolongation and inhomogeneity of the action potential, which leads to arrhythmias, based on abnormal impulse propagation as well as sensitizes the heart to the arrhythmogenic effects of catecholamines. At least 5 genes located on chromosomes 11, 7,3,4 and 21 have been identified with aberrations in encoding for cardiac ion channels in families with LQTS. Romano-Ward syndrome, the autosomal dominant LQTS is sub classified in to LQT 1, which is the commonest form of LQTS and where the mutation is in the gene KVLQT1 on chromosome 11, LQT 2 in HERG on chromosome 7, LQT 3 in SCN5A on chromosome 3, LQT 4 in chromosome 4 and LQT 5 with mutation in Min K on chromosome 21. Jervell-Lange-Nielsen syndrome, the autosomal recessive LQTS is due to mutation in KVLQT1 on chromosome 11 and is associated with deafness. The main diagnostic criteria has been a prolonged QT on ECG but it is to be noted that genetically proven LQTS patients may have normal QT interval. Other diagnostic criteria include a slow heart rate for age, T wave alternans and abnormal T wave morphology. Patients usually present with syncope or cardiac arrest precipitated by emotional or physical stress. The syncope is usually due to "torsade de pointes" frequently degenerating in to ventricular fibrillation. Neonates with LQTS may be identified by a partial AV block and slow heart rate due to prolonged repolarisation. History of syncope, virulent family history, T wave alternans, a QTc exceeding 0.54 s and associated AV block in neonates carry a higher risk. The mortality among untreated symptomatic patients reaches 70% within 15 years from the time of the first syncopal episode. Treatment options include beta-blockade, other anti arrhythmic drugs, stellate ganglionectomy and automatic implantable cardiac defibrillator with marked improvement in survival [13].

***Hypertrophic Obstructive Cardiomyopathy*** (HOCM) is much less prevalent in children than in adults. A Japanese screening programme estimates the prevalence in children at 1 in 15000 compared to an adult prevalence rate of 1 in 500. The overall risk of SCD in HOCM is low and is around one per million in the age specific population under 20 years. The mechanism of SCD in HOCM is believed not to be due to an arrhythmia but ventricular fibrillation has been implicated in some [14].

## Post operative Arrhythmias

***Junctional Ectopic Tachycardia*** (JET) is the commonest (22%) arrhythmia seen in the immediate post-operative period following intracardiac repair mostly following Tetralogy of Fallot correction. The etiology is thought to be due to resection of right ventricular outflow tract (RVOT) muscle bundles, relief of RVOT obstruction through the right atrial approach and an extended bypass time. The risk factors predisposing to post-operative JET have been identified as lower age and body weight at the time of surgery, higher Aristotle basic score, longer cardiopulmonary bypass time and cross clamp time, use of deep hypothermia and total circulatory arrest [15]. The underlying mechanism is enhanced automaticity of the HIS bundle. ECG typically reveals a narrow QRS tachycardia with a rate between 170-230 beats per minute, atrioventricular dissociation with the ventricular rate faster than the atrial rate. JET usually responds to surface cooling to 34ºC, atrial pacing for AV synchrony, sedation and muscle relaxation to avoid stress and intravenous amiodarone.

***Late post-operative arrhythmias*** are the commonest medical problem encountered after repair of congenital heart defects. The incidence relates to the complexity of the underlying heart defects and the clinical significance depends on the interaction between the arrhythmia and the cardiac status. The arrhythmias are attributed to variety of reasons.

- An underlying electrical substrate associated with specific structural heart defects such as atrial isomerisms, Ebsteins anomaly, corrected TGA.
- Acquired due to the pre-operative natural history as with myocardial fibrosis, cyanotic congenital heart disease
- Due to the surgical suture lines as in Sennings repair
- Due to post-operative haemodynamics as with right atrial dilatation following classical Fontan surgery, TOF repair.

Up to 50% of adult patients who have undergone TOF repair reveal late non-sustained arrhythmias during ambulatory ECG monitoring. Occasionally, symptomatic sustained ventricular arrhythmia is documented in these patients. EP studies have shown these ventricular arrhythmias to be due to re-entry, which requires areas of slow conduction within the myocardium. Pulmonary valve regurgitation seen commonly after TOF repair, results in chronic right ventricular overload and diastolic dysfunction and this has been shown to correlate with QRS prolongation on the ECG. It has also been shown that right ventricular QRS prolongation correlates with a higher incidence of symptomatic arrhythmia and a QRS duration exceeding 180 ms on the resting ECG is the most sensitive predictor of life threatening ventricular arrhythmias [16].

## Bradyarrhythmias

***Congenital complete heart block*** (CCHB) is reported to occur with an incidence of 1 per 25000 live births. When it occurs without an associated underlying heart defect and the diagnosis is made at birth, then the mortality is reported at 15%. The risk of death is significantly less at 3.5% when the diagnosis is made beyond the first year of life. In this study by Michaelsson and Engle, the risk of dying is reportedly greatest in early infancy with half of all deaths occurring early in the first year of life. The overall survival rate was 92% in this group. However, the group with CCHB and associated structural heart defect exhibited a higher mortality of 42.4% in those diagnosed at birth [17]. Another prospective study of 102 CCHB patients in the age range of 16-66 years by Michaelsson and colleagues revealed that 36.2% were either symptomatic with syncope or died and of the 40 patients who were followed up for 30 years or more, only 4 (10%) remained symptom free and without a pacemaker [18]. The risk of sudden death in childhood appears to be small but continues throughout adult life. A low ventricular rate associated with a rapid atrial rate and a large heart on chest X-Ray may be an indicator of those children at risk of dying of heart failure. A prolonged QT interval has been consistently shown to be associated with death. Dewey and colleagues suggested that a mean daytime heart rate less than 50 bpm, particularly in conjunction with junctional exit block as a risk factor for death. Associated tachycardias or a flat junctional response were signs of a poor prognosis.
